# In-house texture measurement using a compact neutron source

**DOI:** 10.1107/S1600576720002551

**Published:** 2020-03-25

**Authors:** Pingguang Xu, Yoshimasa Ikeda, Tomoyuki Hakoyama, Masato Takamura, Yoshie Otake, Hiroshi Suzuki

**Affiliations:** a Materials Sciences Research Center, Japan Atomic Energy Agency, 2-4 Shirakata, Tokai, Ibaraki 319-1195, Japan; b Center for Advanced Photonics, RIKEN, 2-1 Hirosawa, Wako, Saitama 351-0198, Japan

**Keywords:** compact neutron sources, texture measurement, neutron diffraction, texture measurement reliability, instrumental accessibility

## Abstract

In order to improve the instrumental accessibility of neutron diffraction techniques, many emerging compact neutron sources and in-house neutron diffractometers are being developed, even though the precision level of neutron diffraction experiments performed on such instruments was thought to be incomparable with that of large-scale neutron facilities. As a challenging project, the RIKEN accelerator-driven compact neutron source (RANS) was employed here to establish the technical environment for texture measurements, and the recalculated pole figures and orientation distribution functions of an interstitial-free steel sheet obtained from RANS were compared with the results from another two neutron diffractometers well established for texture measurement. These quantitative comparisons revealed that the precise neutron diffraction texture measurement at RANS has been realized successfully, and the fine region division of the neutron detector panel is invaluable for improving the stereographic resolution of texture measurements. Moreover, through selectively using the parts of the obtained neutron diffraction patterns that exhibit good statistics, the Rietveld texture analysis improves the reliability of the texture measurement to a certain extent. These technical research results may accelerate the development of other easily accessible techniques for evaluation of engineering materials using compact neutron sources, and also help to improve the data-collection efficiency for various time-resolved scattering experiments at large-scale neutron facilities.

## Abstract 2

The RIKEN compact neutron source and related neutron diffractometer were successfully upgraded for precise in-house texture measurement, as judged by comparison with two other well established neutron diffractometers. This provides a good example for the technical development and the scientific application of neutron diffraction techniques based on compact neutron sources.

## 1. Introduction   

Neutron diffraction is widely thought of as a powerful probe for texture evaluation of advanced materials and even coarse-grained geological samples (Wenk, 1991 ▸; Wenk *et al.*, 1991 ▸; Brokmeier, 1999 ▸; Jansen *et al.*, 2000 ▸), because its large spot size and high penetration enable one to acquire bulk-averaged orientation information from a polycrystalline sample with a volume of the order of 1 cm^3^ (Vogel, 2013 ▸; Malamud *et al.*, 2014 ▸; Yusuf & Kumar, 2017 ▸; Hayashi *et al.*, 2018 ▸). Other crystalline information, *e.g.* lattice parameters, phase fractions, strains and stresses, may be also extracted through a combined Rietveld analysis (Lutterotti *et al.*, 1997 ▸, 2004 ▸; Wenk *et al.*, 2003 ▸; Xu *et al.*, 2018 ▸). These bulk-averaged textures and related crystalline information are extremely valuable for developing various advanced structural and functional materials (Xu *et al.*, 2015 ▸; Liss *et al.*, 2016 ▸; Yusuf & Kumar, 2017 ▸; Mo *et al.*, 2018 ▸; Takajo *et al.*, 2018 ▸). For example, with the aim of further improving the strength–ductility balance of transformation-induced-plasticity low-alloy steels, the lattice parameters and volume fraction of retained austenite have been precisely measured through a neutron diffraction texture measurement (Xu *et al.*, 2017 ▸; Tomota *et al.*, 2017 ▸). If the effect of residual stresses can be omitted or reasonably considered, the carbon concentration of retained austenite may be evaluated indirectly from the lattice parameters of austenite and ferrite through experiential relationships (Onink *et al.*, 1993 ▸; Sugimoto *et al.*, 2003 ▸; Chen *et al.*, 2006 ▸). With the aim of understanding the structural and magnetic properties and further optimizing the magnetic phase microstructure at the nanoscale, neutron diffraction texture measurements have been employed to investigate permanent magnets (Wroblewski *et al.*, 1999 ▸), morphotropic piezoceramics (Hinterstein *et al.*, 2015 ▸), and other advanced magnetic materials for modern computers, medical instruments, ultrasonic motors, electric generators, telecommunications and transportation (Yusuf & Kumar, 2017 ▸).

In order to obtain good straight and strong neutron beams for precise neutron diffraction measurements, nuclear reactors, proton accelerators or other large-scale neutron facilities are usually thought essential (Liss, 2017*a* ▸,*b* ▸; Argyriou & Allen, 2018 ▸). A huge budget is required to cover the establishment of the related hardware and software, the general maintenance, and the ordinary running costs. As a result, the number of available neutron beam instruments across the world is quite limited (Jansen *et al.*, 2004 ▸). Though some rapid measurement platforms based on robotic sample-exchange systems (Hoshikawa *et al.*, 2009 ▸; Reiche & Vogel, 2010 ▸; Brokmeier *et al.*, 2011 ▸) have been attempted, acquiring the necessary neutron beam time is still highly competitive. If no progress is made in increasing the availability of neutron diffractometers, such high competition will continue for several decades because of the gradually increasing application needs. These applications may include averaged texture optimization for developing low-carbon strip-cast steels (Xu *et al.*, 2006 ▸), heavy-gauge shipbuilding steel plates (Hase *et al.*, 2016 ▸; Nishimura *et al.*, 2007 ▸; Nishimura & Takeuchi, 2014 ▸), delayed fracture-resistant ultrahigh streel plates (Xu *et al.*, 2019 ▸), lean duplex stainless steels with low Ni content (Takahashi *et al.*, 2020 ▸) and other formable high-strength lightweight metallic materials. Moreover, high statistically averaged textures are also expected during the numerical simulation and process optimization for press-forming behavior of high-strength metallic materials (Delannay *et al.*, 2006 ▸; Takamura *et al.*, 2013 ▸; Choi *et al.*, 2013 ▸; Hama *et al.*, 2015 ▸). The research and development significance of alternative compact neutron sources has already been emphasized by the JFE Techno-Research Corporation (Sato *et al.*, 2017 ▸), because, as a common measurement tool within manufacturing industries, the neutron diffraction technique will accelerate the innovation of steel production technology through various strong industry–academic community links.

Fortunately, many compact neutron source facilities are being developed around the world (Anderson *et al.*, 2016 ▸), and the Union for Compact Accelerator-Driven Neutron Sources has also been organized to promote technical information exchange about small accelerator-based neutron sources and related neutron scattering techniques. Compared with the large-scale neutron source facilities, these compact neutron source facilities (Anderson *et al.*, 2016 ▸), such as the RIKEN accelerator-driven compact neutron source (RANS) (Yamagata *et al.*, 2015 ▸; Otake *et al.*, 2017 ▸; Otake, 2018*a* ▸,*b* ▸) and the Jülich High-Brilliance Neutron Source (Rücker *et al.*, 2016 ▸), usually have a shorter flight beam path, a larger beam divergence angle, a lower beam power output and higher background noise. Consequently, the most urgent technical problem is how to realize precise neutron diffraction measurements using a weak neutron beam facility.

Recently, RANS has been developed as an in-house multipurpose neutron facility for nondestructive inspection (Otake *et al.*, 2017 ▸; Ikeda *et al.*, 2017 ▸), steel corrosion imaging (Taketani *et al.*, 2017 ▸) and the volume fraction analysis of retained austenite (Ikeda *et al.*, 2016 ▸, 2018 ▸). Because the texture optimization of advanced materials has attracted wider attention for better strength–ductility balance and functional performance, we have attempted to establish a neutron texture measurement environment at RANS through various technical optimizations. A well evaluated interstitial-free (IF) steel sheet sample (Xu *et al.*, 2008 ▸) was employed here as a typical texture material for reference, and the reliability of the texture measurement was evaluated through carefully comparing the bulk textures obtained at RANS and two other well established time-of-flight neutron diffractometers. Such technical research was carried out to accelerate the establishment of other easily accessible techniques for evaluation of engineering materials using neutron diffraction and to promote the wider application of compact neutron diffraction techniques.

## 2. Experimental procedures   

### 2.1. Low-energy nuclear reaction for producing neutrons   

In contrast to the conventional neutron production method using the thermal chain fission reaction in a ^235^U or ^239^Pu nuclear reactor or using a spallation reaction driven by a high-energy proton accelerator (Anderson *et al.*, 2016 ▸), RANS has been developed to produce neutrons through the nuclear reaction between the beryllium metallic target 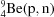 and low-energy protons (Hawkesworth, 1977 ▸), as follows:
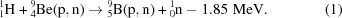
Here, the negative reaction energy means that this nuclear reaction is endothermic, and it requires a net energy input with a critical energy of 1.85 MeV. However, considering the initial kinetic energy of the protons, the practical threshold energy is *E*_th_ = 2.06 MeV (Hawkesworth, 1977 ▸; Yamagata *et al.*, 2015 ▸; Anderson *et al.*, 2016 ▸; Hirota, 2018 ▸).

Generally, a higher net release of nuclear energy corresponds to stronger radiation, especially for the thermal fission reaction at a nuclear reactor. So, the low-energy nuclear reaction for neutron production at RANS simplifies the radiation shielding of the target station and other equipment (Yamagata *et al.*, 2015 ▸; Ma *et al.*, 2018 ▸). The RANS incident proton energy is 7 MeV, so the total neutron yield is ∼10^12^ n s^−1^ at a full-power averaged proton beam current of 100 µA (Otake, 2018*a* ▸).

### 2.2. RIKEN accelerator-driven compact neutron source   

**Figure 1:**
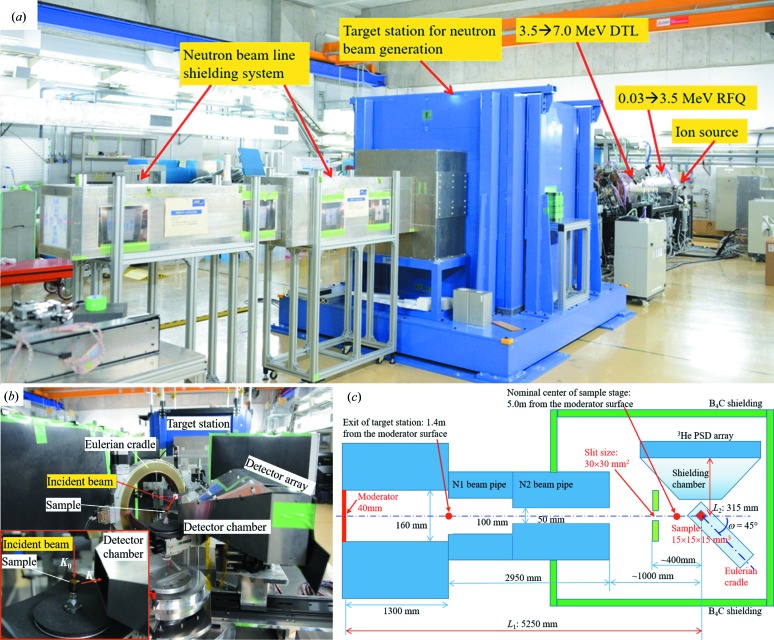
Neutron diffraction texture experiment at RANS. (*a*) Overview. (*b*) View of the sample setup. The rolling direction of the reference sample is parallel to the mouth-opening direction of the Eulerian cradle in the horizontal plane. (*c*) Geometric parameters for the neutron flight paths (*L*_1_, *L*_2_) and the incident beam slit size (30 × 30 mm).

Fig. 1 ▸(*a*) shows the RANS system, about 15 m total length and 25 ton total weight, consisting of an ion source (30 keV), linear proton accelerators [0.03 → 3.5 MeV radio-frequency quadrupole (RFQ) and 3.5 → 7.0 MeV drift-tube linac (DTL))], a target station for neutron beam generation, movable neutron guide tubes, a sample stage and a neutron detector. Such compact equipment helps the rapid exchange of experimental setup between neutron diffraction, neutron scattering and neutron imaging.

In the target station, 50 mm-diameter 0.3 mm-thick 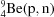 metal is employed as the target film, whose thickness is sufficient to slow down the proton beam. A 4.5 mm-thick vanadium plate is used as the backing material of the target beryllium film, because vanadium has the highest hydrogen diffusional coefficient and the highest hydrogen embrittlement resistance. This vanadium plate is further cooled by water flowing in a 5 mm-thick titanium cavity (Yamagata *et al.*, 2015 ▸). Reaction (1) generates fast neutrons with a maximum energy of about 5 MeV and with a flux peak at around 1.5 MeV (Otake *et al.*, 2017 ▸). These fast neutrons are further slowed down by using a 40 mm-thick polyethyl­ene moderator (the moderator may be replaced with 20 or 60 mm-thick moderators, according to experimental needs) to obtain the thermal neutrons. Cubic graphite reflector blocks (of side length 400 mm) surrounding the polyethyl­ene moderator are employed to increase the low-energy neutron flux.

Fig. 1 ▸(*b*) gives an overview of the experimental layout for the neutron diffraction texture measurement. The movable sample stage is surrounded by movable 5 mm-thick B_4_C rubber shielding plates with reduced background radiation disturbance. The incident neutron flight path (*L*_1_) from the sample center to the surface of the polyethyl­ene moderator and the diffracted neutron flight path (*L*_2_) from the sample center to the flattened neutron detector array may be freely adjusted together with the multiple-purpose sample stages for a specific neutron scattering experiment. Fig. 1 ▸(*c*) illustrates the geometric parameters *L*_1_ = 5250 mm and *L*_2_ = 315 mm employed in this texture measurement. According to our practical experience, these allow us to realize a good balance between high intensity and high time-of-flight resolution (Johnson & Daymond, 2002 ▸). Optimization of the compact neutron source and the moderator system for stronger neutron beam flux and higher time-of-flight resolution is still ongoing.

**Figure 2:**
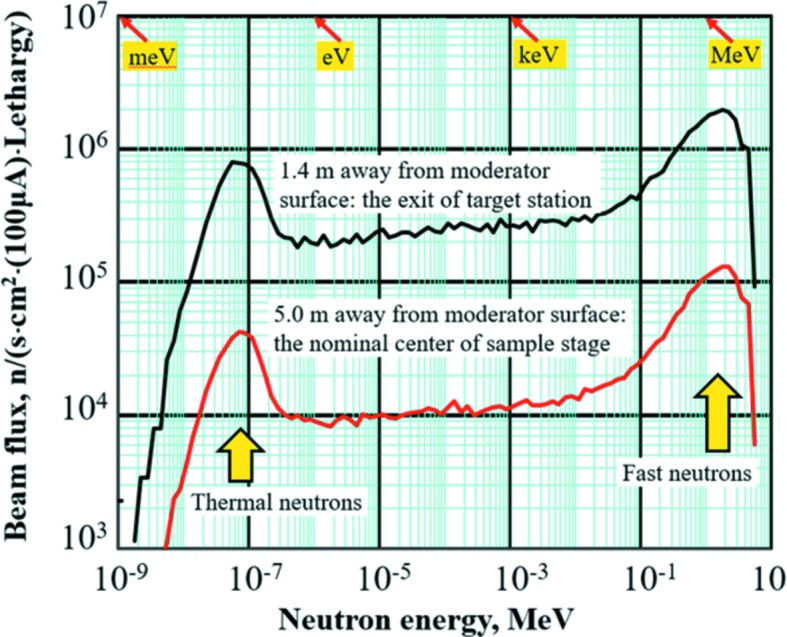
The neutron energy spectra of the RANS pulsed neutron beam at 1.4 and 5.0 m away from moderator surface, simulated by the *PHITS* code (Sato *et al.*, 2013 ▸), expressed in neutrons per second per cm^2^ per lethargy at the RANS full-powder averaged current of 100 µA. The moderator thickness is 4.0 cm, and the neutron lethargy (*i.e*. logarithmic energy decrement) *u* = ln(*E*_0_/*E*) is a dimensionless logarithm of the ratio of the energy of source neutrons to the energy of product neutrons after a collision.

Fig. 2 ▸ shows the neutron energy spectra of fast and thermal neutrons measured at two ‘feature’ positions of the RANS, as marked by red points in Fig. 1 ▸(*c*): (*a*) the exit of the target station and (*b*) the nominal center of the sample stage, 1.4 and 5.0 m from the moderator surface, respectively. The peak energy of the thermal neutrons within an energy range of *E* = 10–360 meV is around 50 meV, suitable for general diffraction experiments (Otake *et al.*, 2017 ▸), corresponding to a maximum beam flux at 1.28 Å within a wavelength range of 2.86–0.48 Å according to the equation λ (Å) = [81.787/*E* (meV)]^1/2^ (Windsor, 1981 ▸). The peak energy of the fast neutrons within an energy range of *E* = 0.1–5.0 MeV is around 2.0 MeV, suitable for fast neutron imaging (Taketani *et al.*, 2017 ▸). The full-power averaged current of the proton beam is 100 µA, where the beam pulse width and the pulse frequency can be adjusted in the ranges 8–180 µs and 10–180 Hz, respectively. During this texture measurement, the averaged current of the proton beam flux was about 32 µA, the beam pulse width was 30 µs, the pulse frequency was 115 Hz and the duty cycle was about 1.3%. Moreover, because of the clear difference in the neutron flight distances of the two feature positions, the beam flux at the nominal center of the sample stage (5.0 m) is lower than that at the exit of the target station (1.4 m), about 4.2 × 10^4^ versus 7.7 × 10^5^ n [s cm^2^ (100 µA) lethargy]^−1^, respectively, for the thermal neutrons and about 1.2 × 10^5^ versus 2.0 × 10^6^ n [s cm^2^ (100 µA) lethargy]^−1^ for the fast neutrons. The RANS thermal neutron flux at the nominal center of the sample stage is about 0.43%, 0.17% of the corresponding values from the TAKUMI neutron diffractometer {about 9.6 × 10^6^ n [s cm^2^ (66 µA)]^−1^ in this paper; the full-power design value will be about 4.8 × 10^7^ n [s cm^2^ (330 µA)]^−1^} and the HIPPO neutron diffractometer {about 2.4 × 10^7^ n [s cm^2^ (120 µA)]^−1^; Wenk *et al.*, 2003 ▸}.

### 2.3. The RANS neutron diffractometer and its 2D neutron detector array   

**Figure 3:**
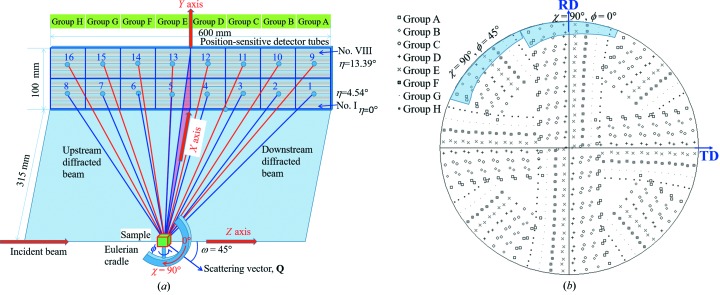
(*a*) Illustration for 16 divided panel regions of the 2D detector array containing eight position-sensitive detector tubes. (*b*) Orientation coverage achieved with the nominal central locations of divided detector panel regions through all 48 sample rotations in the equal-area pole figure projection; the diffraction patterns are summarized into eight bank groups, and the orientation coverages for two sample rotations (χ, ϕ) = (90°, 0°) and (90°, 45°) are marked in light blue.

Fig. 3 ▸(*a*) shows the 2D detector array, consisting of eight Ø12.7 mm × 600 mm position-sensitive detector tubes filled with ^3^He gas at 10 standard atmospheres (1 atm = 101.325 kPa), having a spatial resolution of 10 mm along the tube axial direction and a neutron time-of-flight resolution of several microseconds. The bottom boundary of the detector array is aligned with an initial azimuthal angle η = 0°, where the maximum spanning of azimuthal angle (Δη) is about 17.8°. This detector array is vertically set up along the horizontal direction from *Z* = −300 mm to *Z* = 300 mm, obtaining a scattering angle spanning Δ2θ = 87.2° (or 2θ = 46.4–133.6°). Considering the balance between the diffraction intensity and the time-of-flight resolution of the expected neutron diffraction patterns (Johnson & Daymond, 2002 ▸), the detector array panel was divided into 16 panel regions [2 (vertical) × 8 (horizontal)], and their azimuthal angles for the geometrical centers of the bottom and top regions were 13.4 and 4.5° (or, the azimuthal angle span is Δη = 17.8°), correspondingly.

For the neutron diffraction pattern from each divided region, the following equations were employed to prepare the necessary instrumental parameters according to the de Broglie wavelength equation and the Bragg diffraction law (Jorgensen *et al.*, 1989 ▸):
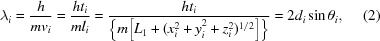


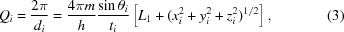
where *v*, *t* and λ are the flight speed, the flight time and the wavelength of the incident neutrons, respectively, *h* is the Planck constant, *m* is the neutron mass, *l* is the total flight path of the neutrons, *Q* is the momentum transfer, *d* is the lattice plane spacing, usually abbreviated as the *d* spacing, and (*x_i_*, *y_i_*, *z_i_*) and 2θ*_i_* are the coordinates and the scattering angle of the geometrical center of the panel region No. *i*. Here *i* = 1, 2,…, 16 and *x_i_* = *L*_2_ = 315 mm. The resolution of the time-of-flight neutron diffraction can be calculated as follows (Windsor, 1981 ▸; Jorgensen *et al.*, 1989 ▸):
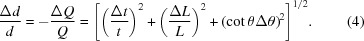
Accordingly, a small wavelength interval (Δλ = 0.005 Å) corresponding to a binning time width (Δ*t* ≃ 10.63 µs) was employed here for each divided panel region to avoid any additional increase of the instrumental error.

A pure body-centered cubic (b.c.c.) Fe powder sample filled in a Ø8 mm × 60 mm vanadium can (S), a similar empty vanadium can (C), a Ø15 mm × 40 mm vanadium–nickel alloy sample (V) and the no-sample background (B) were each measured for 60 min, and the following ratio involving the sample cross section (Windsor, 1981 ▸) was employed to correct the intensity distribution *I*(λ) or *I*(*t*) of the neutron diffraction pattern from the divided panel region No. *i* (*i* = 1, 2,…, 16):
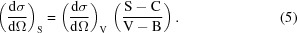



### 2.4. Texture measurement and Rietveld texture analysis   

70% cold-rolled and annealed IF steel with composition 0.0018 C–0.01 Si–0.17 Mn–0.013 P–0.006 S–0.01 Cu–0.01 Ni–0.02 Cr–0.003 V–0.03 Ti–0.026 Nb–0.033 Al_solute_–0.0014 N_total_ (mass%) (Xu *et al.*, 2008 ▸) was employed to prepare a rounded-edged 15 × 15 × 15 mm reference sample and examine the reliability of texture measurement at RANS. The sample was chosen to be larger than the usual 10 × 10 × 10 mm reference sample for large-scale neutron facilities, because the long measurement time due to the weak beam flux at RANS may be shortened through using a larger gauge volume.

**Figure 4:**
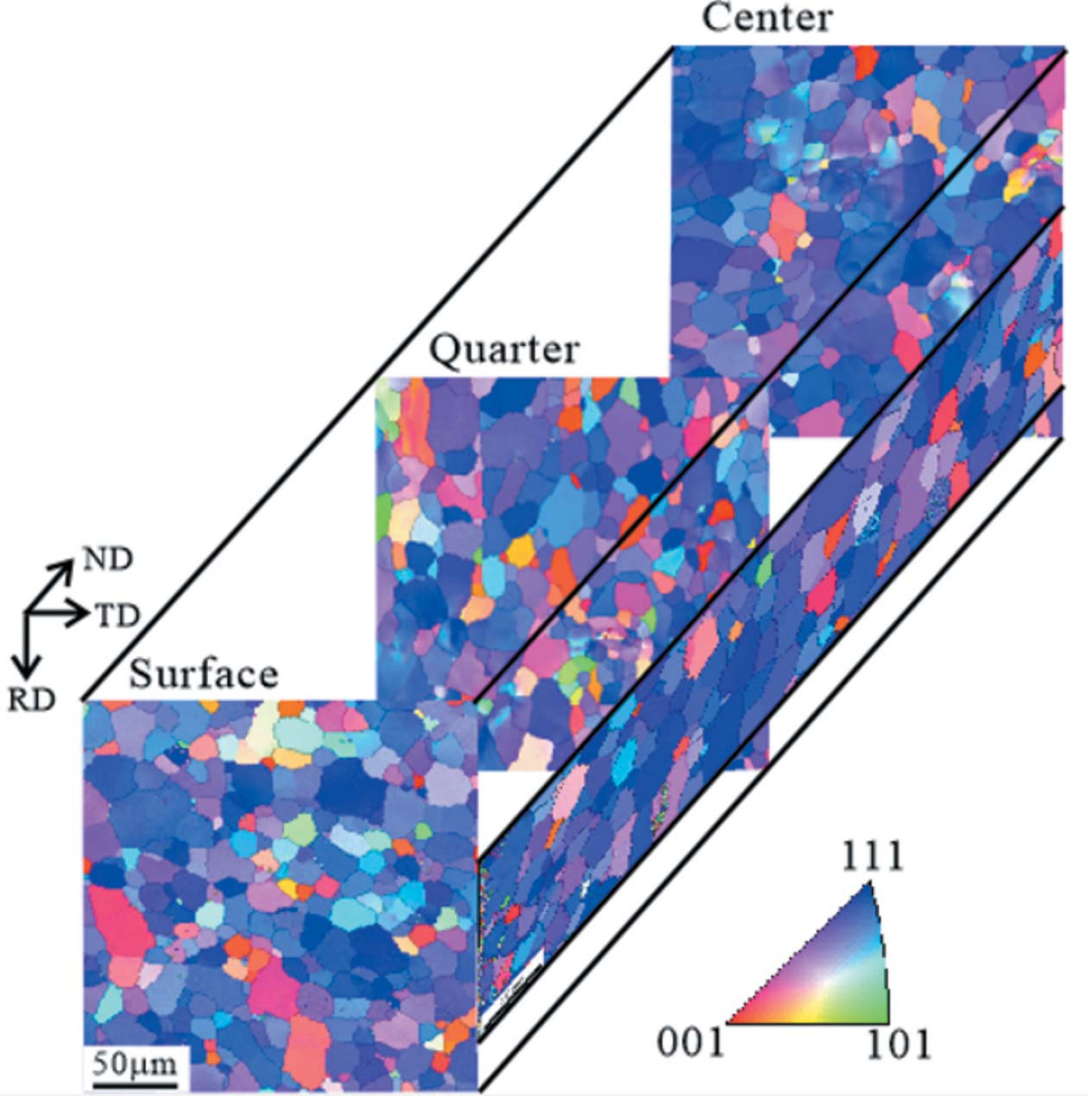
Crystallographic orientation gradient distribution from surface to center of the interstitial-free steel. The inverse pole figure maps for different sample sections are presented here in a three-dimensional way through referring to the normal direction of the steel sheet. ND: normal direction; RD: rolling direction; TD: transverse direction.

**Figure 5:**
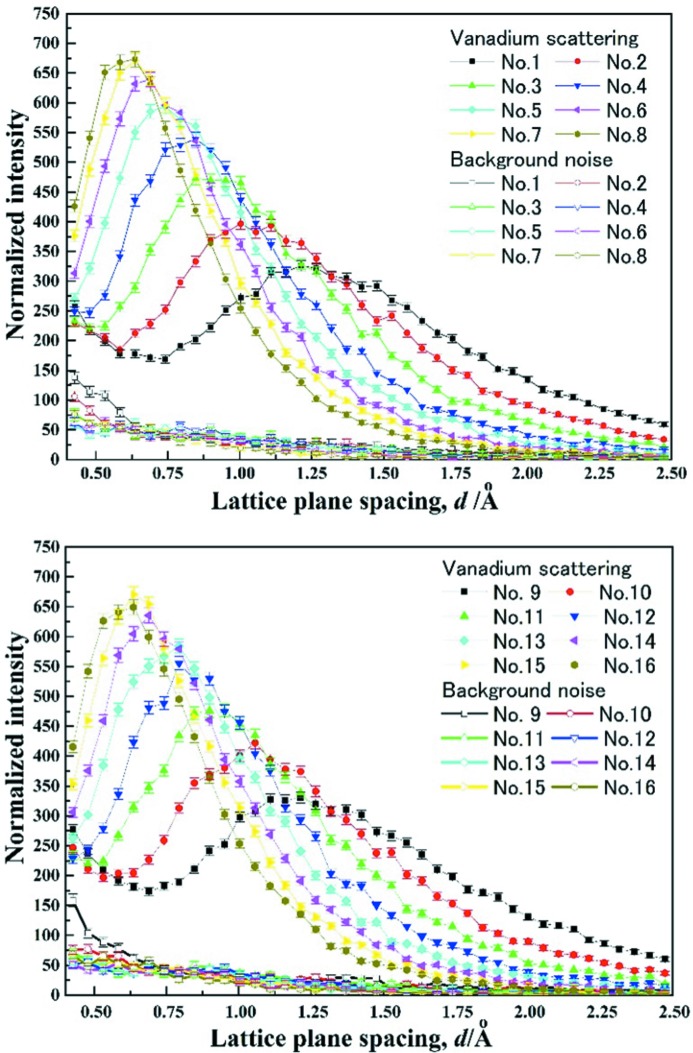
Incoherent vanadium and background scattering characteristics of all 16 divided panel regions, measured with a Ø15 mm × 40 mm vanadium–nickel alloy and without any sample, respectively.

After section fine-cutting of the IF steel sheet and an electrochemical polishing treatment of the new surfaces, the grain orientation characteristics were observed using electron backscatter diffraction (EBSD) with a Hitachi S-4300SE field-emission scanning electron microscope. Fig. 4 ▸ shows the 3D grain orientation distribution characteristics [previously represented in Figs. 5 and 6 of Xu *et al.* (2008 ▸)]. Through the inverse pole figure maps referring to the normal direction (ND), it is found that the IF steel sheet has a strong {111}〈*hkl*〉 fiber recrystallization texture together with a weak {001}〈*hkl*〉 texture component, and the ferrite grains in the surface layer are a little finer than those in the center layer.

During the neutron diffraction measurements of the IF steel sample, the incident beam slit size was set to 30 × 30 mm to ensure that the sample was completely bathed in the neutron beam. The IF steel sample was measured with 48 (χ, ϕ) rotations by using the Eulerian cradle, where χ and ϕ are, respectively, the angles between the ND and RD (rolling direction) of the IF steel sample and the cradle-dependent nominal scattering vector **Q** in the horizontal plane. When the ND is set up along the vertical direction then χ = 90°; when the ND is along the horizontal direction then χ = 0° [Fig. 3 ▸(*a*)]. Fig. 3 ▸(*b*) shows that the orientation coverage for all the diffraction patterns from 48 rotations is more than 90% of the stereographic angle of a complete pole figure; the orientation coverages for two (χ, ϕ) rotations are marked with light blue (Takajo & Vogel, 2018 ▸). The collection time for neutron diffraction patterns of the IF steel sample at each step was 5 min, and the total time including the sample rotation was about 300 min.

In future, another two neutron detector arrays will be added to RANS for a larger azimuthal angle span (Δη ≃ 43.6°) and the proton beam current will be increased. Through the reduced sample rotations and the shortened neutron collection time for each sample orientation, the RANS neutron texture measurement may be finished within 60 min for the large-sized IF steel reference sample.

All of the divided panel and intensity-corrected neutron diffraction patterns (Nos. 1–16) of the IF steel sample obtained from 48 rotations were simultaneously refined using the *Materials Analysis Using Diffraction* (*MAUD*) software (Lutterotti *et al.*, 1997 ▸), and Rietveld texture analysis was carried out using the extended Williams–Imhof–Matthies–Vinel (E-WIMV) texture algorithm implemented in *MAUD* (Matthies & Vinel, 1982 ▸; Lutterotti *et al.*, 2004 ▸), as shown in the pole figure [Fig. 3 ▸(*b*)], through eight different bank groups according to their scattering angles 2θ: (*a*) 51.9–52.6° for Group A (No. 1 and No. 9); (*b*) 62.8–63.4° for Group B (No. 2 and No. 10); (*c*) 73.7–74.1° for Group C (No. 3 and No. 11); (*d*) 84.6–84.7° for Group D (No. 4 and No. 12); (*e*) 95.4–95.3° for Group E (No. 5 and No. 13); (*f*) 106.3–105.9° for Group F (No. 6 and No. 14); (*g*) 117.2–116.6° for Group G (No. 7 & No. 15); (*h*) 128.1–127.4° for Group H (No. 8 and No. 16). During the Rietveld texture analysis, the crystallographic orientations were fitted at an orientation distribution function (ODF) resolution of 5°, sample symmetry was not presumed, and the pre-setup orientation angle (χ_0_, ϕ_0_) and the geometrical center (*x*_0_, *y*_0_, *z*_0_) of the sample were alignment adjusted and refined automatically.

In order to evaluate the reliability of the RANS texture measurement technique, the Rietveld texture analysis results of the same IF steel sample based on two well established time-of-flight neutron diffractometers were employed here for comparison: one was the HIPPO neutron diffractometer at the Los Alamos Neutron Source Center (LANSCE), Los Alamos National Laboratory (Wenk *et al.*, 2003 ▸), and the other was the TAKUMI engineering diffractometer at J-PARC (Xu *et al.*, 2018 ▸). During the HIPPO neutron experiment in December 2009, the sample was measured through four rotations, ω = 0, 45, 67.5 and 90°, and the neutron diffraction patterns from the scattering angles 2θ = 144, 90 and 39° were collected for 10 min per rotation. During the TAKUMI neutron experiment in February 2013, the same sample was measured through 120 rotations to obtain the complete pole figures in high stereographic resolution. In order to compare the texture quantitatively, the corresponding ODFs were calculated using the spherical-harmonic function series expansion method (Bunge, 1982 ▸).

It should be mentioned that HIPPO has since been upgraded through adding the new 2θ = 120 and 60° detector bank groups to acquire in total 53 diffraction patterns by each sample rotation (Reiche *et al.*, 2012 ▸), so that three rotations may realize about 90% pole figure coverage of sample orientations (Takajo & Vogel, 2018 ▸). Recently, comparable measurements at TAKUMI using other texture samples confirmed that 19 rotations using a pseudo-equal-area scanning routine (Gnäupel-Herold & Creuziger, 2011 ▸) are practicable to obtain a good stereographic coverage of the 1/4 pole figure for reliable texture analysis of multiphase steels and other high-crystal-symmetry materials.

## 3. Results and discussion   

### 3.1. Stereographic region division and instrumental scattering characteristics   

Fig. 5 ▸ gives the incoherent vanadium and background scattering characteristics collected from all the divided panel regions (Nos. 1–16). The vanadium scattering patterns No. 1 and No. 9 of Bank Group A at 2θ = 51.9–52.6° show a typical intensity distribution combining thermal neutrons at a lattice plane spacing range of *d* = 1.0–2.0 Å with epithermal neutrons at *d* < 0.5 Å (Windsor, 1981 ▸). The scattering patterns No. 8 and No. 16 of Bank Group H at 2θ = 128.1–127.4° show a strong neutron intensity distribution around *d* = 0.6 Å, and the local vanadium scattering intensity at *d* > 1.5 Å is almost comparable to the background intensity. The other diffraction patterns of the Bank Groups B–H show a gradient transition between these extreme intensity distributions. This clear difference in scattering characteristics between various bank groups reveals that an appropriate region division of the detector panel(s) is essential to utilize the neutron diffraction patterns effectively for a compact neutron source.

**Figure 6:**
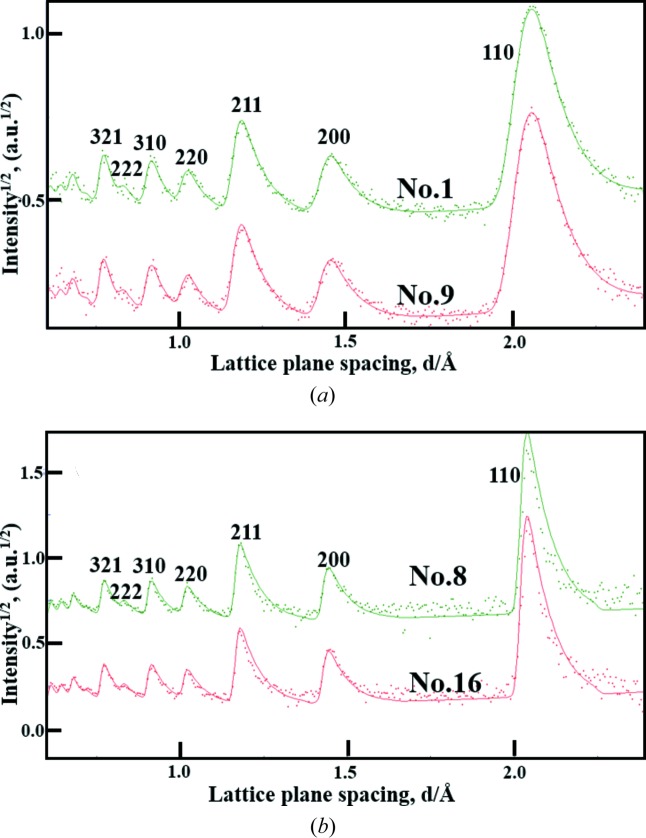
Neutron diffraction patterns of the pure b.c.c.-Fe powder sample filled in a Ø10 mm × 60 mm vanadium can. The measured patterns after various corrections are shown as discrete points, and their fitted curves obtained using the *MAUD* software are shown as solid lines. (*a*) Diffraction patterns No. 1 and No. 9 of Group A at 2θ = 51.9–52.6°. (*b*) Diffraction patterns No. 8 and No. 16 of Group H at 2θ = 128.1–127.4°.

Fig. 6 ▸ shows that the neutron diffraction patterns of the b.c.c.-Fe powder sample (in discrete points) are appropriately corrected and Rietveld refined. The 110 diffraction peak of the measured diffraction patterns No. 1 and No. 9 has a relatively larger FWHM in comparison with that of the measured diffraction patterns No. 8 and No. 16, revealing that the high scattering angle leads to a better instrumental resolution Δ*d*/*d* = −Δ*Q*/*Q*. Meanwhile, it is found that for the diffraction patterns No. 1 and No. 9 the diffraction intensity at a lattice plane spacing range of *d* < 0.8 Å is at a lower statistical precision because of the corresponding local strong background intensity; for the diffraction patterns No. 8 and No. 16, the diffraction intensity at *d* > 1.5 Å is at a lower statistical precision owing to the weak incident long-wavelength neutrons. Moreover, through the good Rietveld refinement of the neutron diffraction patterns, shown here as solid lines, the necessary parameters for the time-of-flight/spacing (*t*/*d*) conversion, the peak-shape refinement *etc.* were extracted to prepare the instrumental file for the *MAUD* texture analysis together with the geometrical parameters of all the panel regions including the scattering angles (2θ), the azimuthal angles (η) and the flight paths (*L*_2_).

### 3.2. Texture analysis of cold-rolled and annealed IF steel   

**Figure 7:**
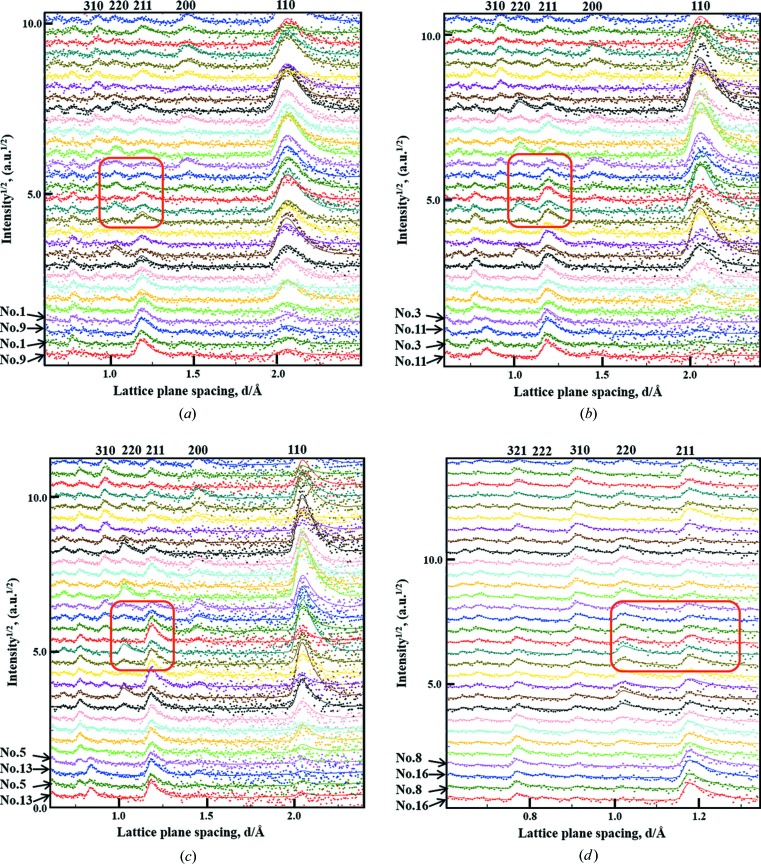
Partial neutron diffraction patterns of the IF steel sample and their partially Rietveld refined patterns: (*a*) Bank Group A (No. 1 and No. 9, at 2θ = 51.9–52.6°); (*b*) Bank Group C (No. 3 and No. 11, at 2θ = 73.7–74.1°); (*c*) Bank Group E (No. 5 and No. 13, at 2θ = 95.4–95.3°); (*d*) Bank Group H (No. 8 and No. 16, at 2θ = 128.1–127.4°).

Fig. 7 ▸ shows typical neutron diffraction patterns of cold-rolled and annealed IF steel and their Rietveld-fitted results. In general, the Rietveld texture refinement is highly satisfactory considering that the peak intensities in each diffraction pattern are not strong. Even for a same-sample rotation, the diffraction patterns from the neighboring panel regions Nos. 3 and 11 and Nos. 5 and 13 show a clear difference in the diffraction intensities of the 211 and 220 peaks (marked by red frames), suggesting that the panel region division is very valuable for a reliable texture measurement. However, for Group A at a lattice plane spacing range of *d* < 0.8 Å, the background noise is stronger and the deviation of the intensity of the measured diffraction patterns from the Rietveld-refined curves is relatively larger; for Group E at a lattice plane spacing range of *d* > 1.4 Å, the weak incident beam flux results in a relatively large deviation of the intensity of the measured diffraction patterns from the Rietveld-refined curves. Therefore, 11 peak reflections were employed from the diffraction patterns of Bank Groups A–E, and only nine peak reflections were employed from the diffraction patterns of Bank Groups F–H, hereafter referred to as ‘unequal *d* ranges’ (*i.e.* for Groups A–E: *d* = 0.6–2.4 Å; for Groups F–H: *d* = 0.6–1.4 Å). The slightly different case of using 11 peak reflections from Bank Groups A–H is referred to as ‘equal *d* ranges’, or *d* = 0.6–2.4 Å.

**Figure 8:**
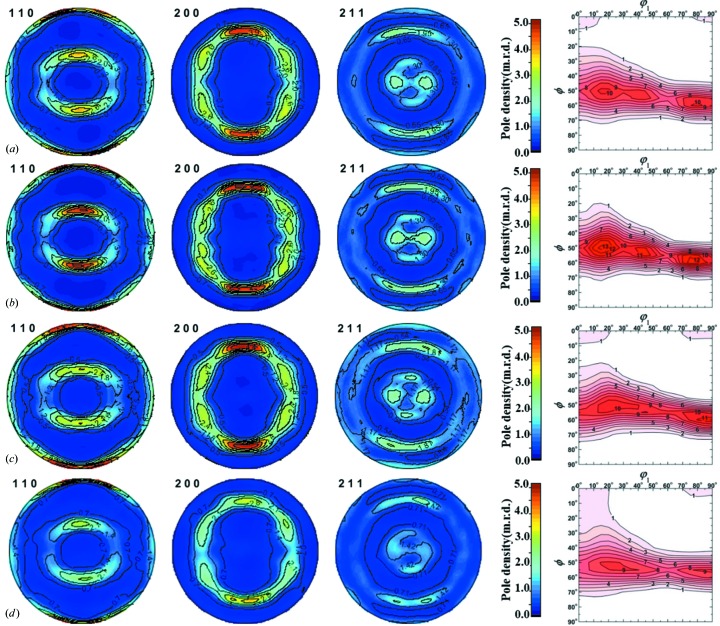
Recalculated pole figures (in equal-area projection, left) and orientation distribution functions (φ_2_ = 45° section, right) of the IF reference steel obtained from the measured neutron diffraction patterns through the Rietveld texture analysis: (*a*) from RANS using unequal *d* ranges (for Groups A–E: *d* = 0.6–2.4 Å; for Groups F–H: *d* = 0.6–1.4 Å), *I*_max_ = 4.85 m.r.d. (multiples of a random distribution) in pole figures, *f*_max_ = 10.89 m.r.d. in ODF, texture index *F*2 = 6.73; (*b*) from RANS using equal *d* ranges (*d* = 0.6–2.4 Å), *I*_max_ = 5.23 m.r.d. in pole figures, *f*_max_ = 13.91 m.r.d. in ODF, texture index *F*2 = 7.51; (*c*) from TAKUMI using equal *d* ranges (*d* = 0.6–2.4 Å), *I*_max_ = 5.19 m.r.d. in pole figures, *f*_max_ = 11.76 m.r.d. in ODF, texture index *F*2 = 7.02: (*d*) from HIPPO using equal *d* ranges (*d* = 0.6–2.4 Å), *I*_max_ = 5.24 m.r.d. in pole figures, *f*_max_ = 9.30 m.r.d. in ODF, texture index *F*2 = 6.61.

Fig. 8 ▸ shows the recalculated pole figures in equal-area projection obtained by using the diffraction patterns measured from RANS, TAKUMI and HIPPO, together with the φ_2_ = 45° ODF sections calculated by using the series expansion texture calculation method at an expansion series of *L*_max_ = 32. Moreover, the corresponding values of the texture index *F*2, *i.e*. the integral of the square of the texture function *f*(*g*) (Bunge, 1982 ▸), are given here for reference. The preferred orientations in the pole figures in Fig. 8 ▸(*a*) from RANS using ‘unequal *d* ranges’ are similar to those in Fig. 8 ▸(*c*) from TAKUMI and Fig. 8 ▸(*d*) from HIPPO using ‘equal *d* ranges’, including good orientation symmetry, no ghost orientations and almost consistent pole density distribution for typical texture orientations, suggesting the RANS technical environment for texture measurement has been established reliably. In contrast, Fig. 8 ▸(*b*) shows an unexpected stronger density distribution in the 110 pole figure, mostly due to the uncertainty in the intensities of the diffraction patterns of Banks F–H at *d* > 1.4 Å. Moreover, from the φ_2_ = 45° ODF sections in Figs. 8 ▸(*a*), 8 ▸(*c*) and 8 ▸(*d*), the strong {111}〈uvw〉 γ fiber at around φ_1_ = 0–90°, ϕ = 55°, φ_2_ = 45° together with a weak {001}〈110〉 rotated-cube component at around φ_1_ = 0°, ϕ = 0 and 90°, φ_2_ = 45° confirms the crystallographic orientation characteristics observed from the EBSD mapping results (Fig. 4 ▸) at a higher statistical level. Fig. 8 ▸(*b*) shows overestimated γ-fiber and underestimated rotated-cube components, which is almost unacceptable as a high-statistical-precision measurement result.

Since HIPPO is a well established neutron diffractometer for texture measurement (Wenk *et al.*, 2003 ▸), which has been widely employed in many academic studies, and TAKUMI has been established for high-stereographic-resolution texture measurement (Xu *et al.*, 2018 ▸), the above results reveal that, at the least, RANS and the related texture measurement environment are satisfactory for the quantitative texture measurement of steels and other high-symmetry materials. For RANS texture analysis in equal *d* ranges, the reliability of the texture measurement may be improved through longer neutron collection times, and the texture measurements of some complicated materials including titanium alloys and magnesium alloys may be realized. Moreover, because the panel region division at RANS enables us to acquire many concurrent neutron diffraction patterns with good statistics, Rietveld texture analysis in unequal *d* ranges is extremely valuable for a compact neutron source such as RANS.

On the other side, since the same IF steel was measured at HIPPO for 10 min per rotation and at TAKUMI for 0.5 min per rotation, the reliability of the RANS texture measurement from the weak diffraction patterns reveals that the neutron collection time per rotation at HIPPO, TAKUMI and other large-scale neutron facilities could be further shortened to provide more possibility for time-resolved neutron diffraction studies (Onuki *et al.*, 2016 ▸).

## 4. Conclusions and prospect   

In order to improve the instrumental accessibility of the neutron diffraction technique and provide a rapid response to the on-site research needs for advanced metallic materials, the quantitative texture measurement technique was established at RANS. The fine division of the diffraction detector panel ensured the high stereographic resolution of time-of-light neutron diffractograms, and the concurrent Rietveld full-profile refinement of all obtained neutron diffractograms decreased the necessary sample rotations to a certain extent.

All of the 768 neutron diffraction patterns of a cold-rolled and annealed IF steel sample collected through 48 rotations were Rietveld texture analyzed through selectively using parts of the obtained neutron diffraction patterns that exhibited good statistics, and the obtained pole figures were almost consistent with the corresponding results from two well equipped neutron diffraction texture measurement instruments, TAKUMI at J-PARC, Japan, and HIPPO at LANSCE, USA, revealing that the precise neutron diffraction texture measurement at RANS has been realized successfully.

Such technical progress using a compact neutron source to realize precise neutron diffraction texture measurement suggests that we are entering a new era of neutron scattering technology and its engineering applications, and that RANS-like compact neutron sources will be widely used together with in-house neutron scattering facilities for higher instrument accessibility.
